# pH-Control in Aptamer-Based Diagnostics, Therapeutics, and Analytical Applications

**DOI:** 10.3390/ph11030080

**Published:** 2018-08-26

**Authors:** Micaela Belleperche, Maria C. DeRosa

**Affiliations:** Department of Chemistry and Institute of Biochemistry, Carleton University, Ottawa, ON K1S5B6, Canada; micaelabelleperche@cmail.carleton.ca

**Keywords:** aptamer, pH-responsive, drug delivery, biosensors, DNA nanotechnology, i-motif, triplex

## Abstract

Aptamer binding has been used effectively for diagnostics, in-vivo targeting of therapeutics, and the construction and control of nanomachines. Nanostructures that respond to pH by releasing or changing affinity to a target have also been used for in vivo delivery, and in the construction of sensors and re-usable nanomachines. There are many applications that use aptamers together with pH-responsive materials, notably the targeted delivery of chemotherapeutics. However, the number of reported applications that directly use pH to control aptamer binding is small. In this review, we first discuss the use of aptamers with pH-responsive nanostructures for chemotherapeutic and other applications. We then discuss applications that use pH to denature or otherwise disrupt the binding of aptamers. Finally, we discuss motifs using non-canonical nucleic acid base pairing that can shift conformation in response to pH, followed by an overview of engineered pH-controlled aptamers designed using those motifs.

## 1. Introduction

In recent years, aptamers have become a useful molecular recognition tool, with applications ranging from biosensors, to affinity purification, to integration in nanoscale machinery [1,2,3]. Aptamers are synthetic nucleic acid structures with the ability to bind ligands including proteins, small molecules, whole cells, and carbohydrates [4]. Aptamers can act as recognition elements, filling many of the same roles as antibodies; however, aptamers have several advantages over antibodies that make them attractive for therapeutic and biotechnological applications [4]. These include ease of chemical modification, improved stability over time and at variable pH or temperature, reversible conformation change in response to stimulus, and quick and reliable synthesis in vitro [5,6]. Because of these properties, much work has been done on expanding the applications of aptamers and combining them with other functional elements [7,8].

pH-responsive elements have also found use in many molecular systems, including in vivo targeting, sensing, and the motor elements of nanomachines [8,9,10]. Many biological processes are also controlled by pH [9]. One advantage of using pH as a control element is that altering the pH of a solution produces only salts as waste, while other control elements may leave waste compounds that can interfere with later steps or potential re-usability of the system [8,11].

Combinations of these two elements have potential applications in the construction of nanomachines, targeting and delivery of in vivo nanostructures and therapeutics, and control of ligand binding in larger structures such as sensors, diagnostics, and purification columns [8,12]. In general, DNA aptamers are preferred for these applications due to the susceptibility of RNA to base-catalyzed hydrolysis; however depurination at low pH is a concern to be considered with both nucleic acids. This review will discuss some of the previously developed systems that combine DNA aptamers with other pH-responsive elements, as well as the applications of DNA aptamers with passive pH-responsive properties and current research into engineering pH-controlled DNA aptamers. Throughout this review, several distinct terms are used to describe the approach taken in the development of these systems. For example, “passive” systems are those whereby the natural pH responsive properties of the nucleic acid sequence were discovered and utilized, while “engineered” systems include those where known pH responsive motifs have been deliberately or rationally included into a sequence.

## 2. Combination of Aptamers with pH-Responsive Systems for Drug Delivery

Many systems incorporating both aptamers and pH responsive elements have been developed for the targeted delivery of anti-cancer drugs [7,13,14]. Often, chemotherapeutics cause off-target toxicity; directed delivery can increase the effectiveness of the drugs and reduce adverse effects [14]. Biomarkers that are only found, or are overexpressed, on cancerous cells are key targets for drug carriers [15]. Incorporation of aptamers for those proteins can direct the drug delivery mechanisms to bind to the target cell, meaning that the drug is only released close to its target [16]. Aptamer binding of some plasma membrane proteins can cause the bound nanoparticle to be internalized by the cell, along with the drug [15]. Solid tumour tissue has a weakly acidic pH (6.5–7.2) and endosomes or lysosomes within the cell have a pH ranging from 5.0–6.5, compared to the normal tissue pH of 7.4 [10,17]. Systems designed to respond to weakly acidic pH can release their carried drugs only within the tumour microenvironment or only after being internalized into a tumour cell [10,17].

**Figure 1:**
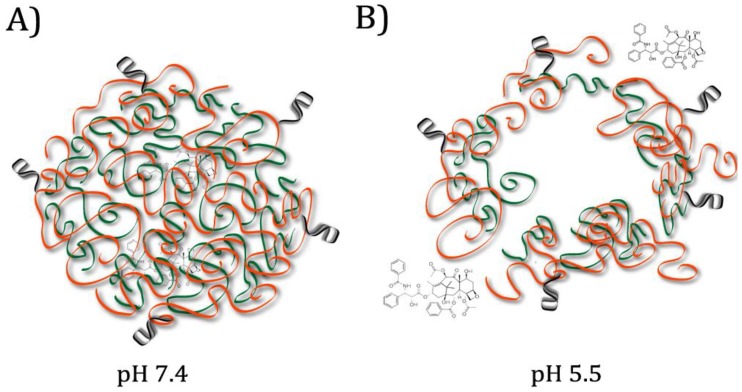
Micelle composed of interior pH-responsive polymer (green) and exterior aptamer-decorated polymer (orange). (**A**) At physiological pH, Hydrophobic Paclitaxel (PTX) is contained in the centre of the micelle. (**B**) In the lysosomes, after aptamer-mediated internalization, low pH causes the pH-responsive polymer to swell and the micelle to break apart, releasing the PTX. Adapted from Zhang et al., 2015 [10].

In many cases, superior targeting is achieved by conjugating aptamers to a separate pH-responsive drug carrier mechanism [16]. One popular method of drug delivery is the encapsulation of a hydrophobic drug within the core of a polymeric micelle; the hydrophilic exterior of the micelle can then be conjugated with targeting ligands or other coatings to increase longevity in the system [14]. In 2015, Zhang et al. designed artificial micelles composed of two polymers: one pH-responsive, and one decorated with aptamers targeting nucleolin, a membrane protein overexpressed on cancer cells that allows internalization when bound by an aptamer [10]. The chemotherapy drug delivered, paclitaxel, is highly hydrophobic and binds to plasma proteins, necessitating a carrier to improve the effectiveness [10]. The pH-responsive polymer degrades at weakly acid pH such as that found in tumour microenvironments, while the nucleolin aptamers allow uptake of the nanoparticles [10]. In vitro tests showed rapid release of the drug from the micelles at pH 5.5 and improved accumulation in the cancerous cells compared to controls without conjugated aptamers [10]. (See Figure 1) Another popular system for drug delivery involves gold nanoparticles [13]. In 2016, Taghdisi et al. designed a system to deliver the anticancer drug daunorubicin using a gold nanoparticle conjugated to aptamers for nucleolin and tyrosine kinase 7 (a biomarker for acute lymphoblastic leukemia T-cells) [13]. Tyrosine kinase 7 was used for nanoparticle targeting, and the nucleolin aptamer was intended to promote internalization through the nuclear membrane [13]. Because the drug was bound to the nanoparticle through electrostatic interactions, protonation at weakly acidic pH caused it to be released more quickly [13]. Many other materials have also been tested for pH-responsive drug release. In 2015, Zhou et al. designed a drug delivery mechanism consisting of a porous calcium carbonate nanoparticle coated with an avidin membrane, conjugated to a biotin-labeled aptamer for protein tyrosine kinase (PTK) [16]. PTK is a biomarker found on human T-cell acute lymphocytic leukemia cells; binding allows the nanoparticle to be internalized [16]. Under the acidic conditions of the lysosome, the calcium carbonate structure dissolves, releasing the doxorubicin stored within the pores [16].

Because some anticancer drugs intercalate with double-stranded DNA, the DNA structure containing an aptamer may act as a drug carrier itself [18]. In 2013, Boyacioglu et al. designed a dimeric DNA aptamer complex targeting prostate-specific membrane antigen, a biomarker overexpressed on prostate cancer cells [18]. The dimeric complex formed a duplex DNA “bridge” containing several target sites for doxorubicin binding [18]. The researchers then covalently attached doxorubicin to its binding sites on the DNA structure, using formaldehyde to create a pH sensitive linker which would release the drug in the lysosomes after internalization [18]. In 2016, Stuart et al. designed a dimeric aptamer complex targeting vitronectin, a biomarker and possible therapeutic target for breast cancer; similarly, they bound the drug to the double-stranded portions of the complex using formaldehyde to create a pH sensitive linker [19]. In 2017, Zhang et al. designed a single-stranded DNA nanoparticle that contained hundreds of repeats of a prostate cancer membrane antigen aptamer, drug loading sites, primer binding sites for amplification, and a pH-sensitive spacer composed of adenine repeats [20]. Doxorubicin was intercalated at the double-helix binding sites, though not covalently bound [20]. Testing showed that target cells bearing the antigen were able to internalize the nanoparticles; at acidic pH, the nanoparticle would then break apart at the pH-sensitive spacers into fragments that would quickly release the drug [20].

Aptamer binding has been used for more than just targeting a drug delivery system. In 2016, Zhang et al. designed a delivery mechanism based on the formation of tight aggregates between PEG polymer, conjugated to doxorubicin with a pH-sensitive bond, and an ATP-binding aptamer. For targeting and internalization, they used an artificial ligand for bradykinin, a protein expressed on breast cancer cells [21]. Lysosomes are known to contain large amounts of ATP; when the aptamer bound to ATP, the interactions between it and the drug-conjugated PEG were disrupted [21]. Notably, while low pH was able to hydrolyze the doxorubicin bond with PEG, the rate of drug release was only increased in the presence of both acidic pH and ATP; otherwise, the unbound aptamer “trapped” the doxorubicin [21]. In the same year, Taghdisi et al. reported the design of a DNA dendrimer nanoparticle using three aptamers: two conjugated to the surface of the particle for targeting and internalization (one nucleolin-binding and one binding MUC1, an abnormal glycoprotein expressed on many cancer cells) and a third, ATP-binding aptamer integrated into the structure of the dendrimer [22]. Epirubicin was intercalated within the dendrimer structure [22]. ATP binding within the lysosomes destabilized the dendrimer, causing it to break apart; additionally, protonation of the drug at acidic pH disrupted its binding to the dendrimer and increased the rate of release [22].

In addition to their role in drug delivery, some aptamers show therapeutic properties themselves [23]. Shen et al. in 2018 published an artificial micelle system designed to carry a therapeutic aptamer [23]. The polymer used as a coating reduces interactions with plasma proteins, while the core polymer is hydrophobic at neutral pH, but at pH 6.0 becomes hydrophilic and causes the micelle to swell and disaggregate [23]. Human epidermal growth factor receptor 2 (HER2) is a receptor tyrosine kinase overexpressed on many cancer cells; a trimeric anti-HER2 aptamer derived from the degradation of HER2 in lysosomes had previously been discovered to kill HER2-overexpressing cells, likely by causing cross-linking, internalization, and degradation of HER2 [23]. The aptamer was encapsulated inside the pH responsive artificial micelles, which were used to improve uptake and release of the therapeutic aptamer intracellularly [23]. In 2017, Zhang et al. designed a polymeric, pH-sensitive micelle for the delivery of both doxorubicin and a therapeutic aptamer for nucleolin [14]. The aptamer, AS1411, had previously been shown to inhibit tumour growth; additionally, it can bind to and trigger internalization into a tumour cell [14]. The micelle carried doxorubicin within its hydrophobic core, while the aptamer was attached electrostatically to the hydrophilic exterior [14]. After the nanoparticle was internalized, the pH-responsive polymer dissociated within the endosome and the drug and aptamer were released [14]. In 2018, Taghdisi et al. described a DNA nanostructure comprised of three strands of the AS1411 aptamer for nucleolin which contained several double-helix sites where doxorubicin could intercalate [24]. The aptamer portions of the structure were able to bind nucleolin and cause the structure to be internalized, at which point protonation of doxorubicin caused the drug to be released [24]. The AS1411 was expected to act as a therapeutic aptamer in combination with the doxorubicin [24].

## 3. Other Applications of Aptamers in pH-Responsive Systems

In addition to drug delivery, aptamers have also been used for the construction of pH sensitive diagnostic probes for tumour imaging. In 2017, Zhou et al. designed a probe using the nucleolin-binding AS1411 aptamer covalently attached to a pH-activatable fluorophore [25]. The probe was internalized into target breast cancer cells through binding to the overexpressed nucleolin on the cell surface; the acidic conditions of the lysosome then caused the probe to increase in fluorescence, releasing a measurably stronger signal [25].

Convenient pH-responsive properties have also been found in systems for which pH-responsiveness was not initially expected. In 2018, Figueroa-Miranda et al. designed an electrochemical impedance biosensor using the aptamer 2008s, which specifically recognizes the plasmodium lactate dehydrogenase protein, a malaria biomarker, without reacting to human lactate dehydrogenase [6]. The aptamer was immobilized on a gold electrode through covalent thiol-gold bonds, after which 6-mercaptohexanol was used to passivate the surface; ferri/ferro-cyanide was used as a redox probe to measure the charge transfer resistance [6]. When protein bound to the aptamer, the researchers found that resistance to current changed depending on both the concentration of protein and the pH: at pH 7.5, the electron transfer resistance decreased proportionately to protein concentration from 0.1 to 100 pM, while at pH 9.5, electron transfer resistance increased proportionately to protein concentration from 100 pM to 10 nM [6]. This was thought to be because of the change in charge as the pH of the solution passes the protein’s pI value of 8.0, attracting (at low pH) or hindering (at high pH) the negatively charged redox probes [6]. By altering the pH, the sensitivity of the sensor was extended over five orders of magnitude [6].

**Figure 2:**
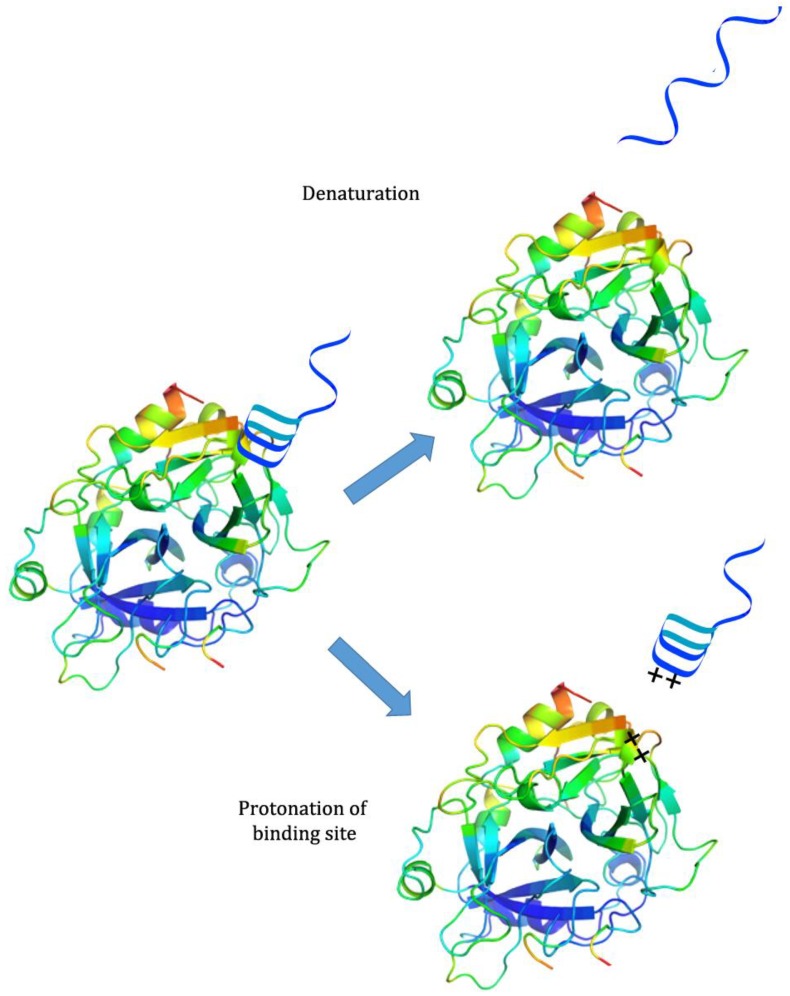
Sources of passive pH-responsiveness in aptamers. **Top**: Extreme pH causes denaturation of the Watson-Crick or non-canonical bonds in the aptamer structure. **Bottom**: Protonation of ligand and/or of binding-site nucleic acids disrupts target binding ability.

While there has been much development of systems incorporating both pH-responsiveness and aptamer binding, there has been less investigation into systems that use pH to control aptamer binding. Because aptamers are generally selected at neutral or physiological pH, many of them partially or completely lose binding capability when exposed to extreme pH; this may be because the aptamer denatures, destroying the binding conformation, or because protonation of the binding site or ligand interferes with binding interactions [26]. However, they are often able to regain their initial binding capability when the pH is returned to neutral [26] (see Figure 2). This property has been exploited for several applications.

## 4. Passively pH-Responsive Aptamers in Purification

In 2015, Bayramoglu et al. described a method for purifying lysozyme from egg whites using a lysozyme-binding aptamer covalently bound to silica particles. The aptamer in question showed the highest affinity for substrate at pH 7, and much lower binding efficiency at more basic or acidic pH [1]. This property was exploited for purification purposes: particles were mixed into a pH 7 sample solution containing lysozyme and removed by centrifugation, after which the lysozyme was desorbed using a pH 2 glycine buffer [1]. The researchers found that over 95% of the adsorbed lysozyme could be eluted using this method [1]. Additionally, the particles proved to be reusable, and were able to adsorb and release over 80% of the initial lysozyme capacity even after 20 cycles [1]. The researchers speculated that the cause of the binding affinity change may have been disruption of noncovalent interactions between the aptamer and lysozyme [1].

Lin et al. described in 2016 an aptamer-functionalized extraction method for toxic poly-chlorinated biphenyls (PCBs) in which a low pH buffer was used for elution [27]. Stainless steel wires coated with a metal-organic framework (MOF) that had been modified to carry a strong positive charge were used to immobilize a G-quartet aptamer for two specific PCBs [27]. At neutral or mildly acidic pH, the G-quartet aptamer was able to specifically capture the desired PCBs when submerged and stirred in sample solution [27]. When the MOFs were removed from solution and washed at pH 3, the structure and/or binding of the aptamer to the substrate was disrupted and the target PCBs could be recovered with high specificity [27]. It was noted that the MOFs could be used repeatedly, indicating that the immobilization of the aptamer was not disrupted by the low pH and the aptamers were able to refold and regain binding capacity when reintroduced into a neutral solution [27].

The following year, Huang et al. used aptamer-functionalized microbeads for magnetic dispersive solid phase extraction, using an aptamer that could simultaneously recognize chloramphenicol, thiamphenicol, and florfenicol [28]. The magnetic microbeads were added to the sample solution and stirred, allowing the aptamer to bind to the substrate amphenicols, after which the beads were collected using a magneton [28]. Like the previous examples, the extraction was performed at the ideal pH for aptamer function (in the case of the aptamer used here, 7.6) and the substrate was desorbed from the aptamer using a buffer of a different pH; unlike the previous examples, the researchers found that for this aptamer, satisfactory recovery was achieved using a buffer of pH 8.5 [28]. The microbeads retained over 80% recovery capacity for all three analytes even after 60 uses [28].

## 5. Passively pH-Responsive Aptamers in Nanoscale Systems

In 2011, Huang et al. noted that an aptamer selected at neutral pH lost affinity to its target at low pH and combined this with a previous observation that the affinity between a DNA aptamer and a graphene oxide surface increased at low pH [26]. The researchers used an adenosine aptamer labeled with a fluorescent marker; when the aptamer was adsorbed to graphene oxide surface, the fluorescent label was quenched [26]. At neutral pH, in the presence of adenosine, the aptamer bound adenosine and desorbed from the graphene oxide surface, resulting in an increase in fluorescence [26]. At pH 3.5, either the affinity for the target or the structure of the aptamer was disrupted, and the aptamer adsorbed to the graphene oxide once again [26]. A loss of about 50% fluorescence was observed after a second usage, but this was attributable to loss of aptamer and graphene oxide during the washing step, rather than any lasting impact on the aptamer performance [26]. In addition to potential reusability, the pH and adenosine responsive system functioned as an AND logic gate: adenosine only caused the system to produce a fluorescent signal if the pH was neutral or high, as the increase in GO binding affinity and loss of target binding affinity at low pH was able to prevent initial release of the aptamer [26].

**Figure 3:**
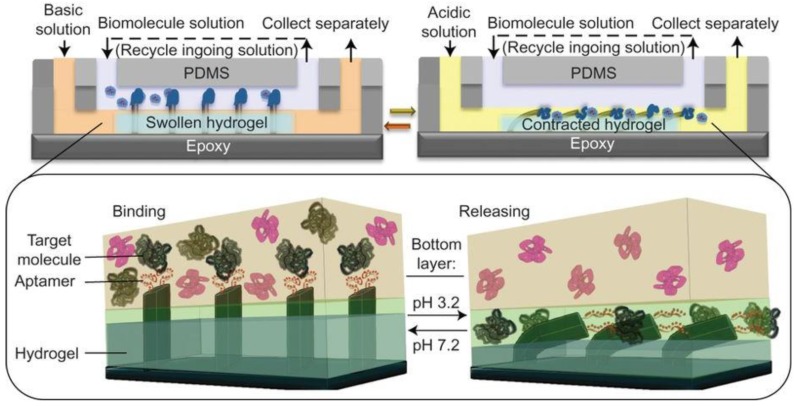
Microfluidic thrombin separation device. In upper laminar flow chamber, fluid is kept at pH 6.3 and solution containing thrombin is run through. Aptamer-decorated fins are attached to a pH-responsive hydrogel in the bottom laminar flow chamber. **Top** and **Bottom Left**: When the lower chamber fluid is at pH 7.2, the hydrogel expands, the fins protrude into the upper chamber, and thrombin is bound. **Top** and **Bottom Right**: When the lower chamber fluid is at pH 3.2, the hydrogel contracts, the fins are pulled into the lower chamber, and the aptamers denature, releasing thrombin. Figure taken with permission from Shastri et al., 2015 [2].

Shastri et al. created a system in 2015 in which the pH-controlled binding of a thrombin aptamer was integrated with a pH responsive hydrogel for the capture and movement of thrombin from one fluid compartment to another [2] (see Figure 3). The pH-responsive hydrogel was located in one of two fluid compartments separated by a constant laminar flow [2]. Epoxy microfins that had been covered with aptamers using PDMS were fixed to the top of the hydrogel, extending into the second flow compartment [2]. The solution passing through the upper compartment was kept at a constant pH of 6.3, observed to be the pH at which the aptamer had the highest binding affinity [2]. Sample containing thrombin was passed through this compartment and bound by the aptamer [2]. When the fluid in the bottom compartment was kept at pH 7.2, the hydrogel expanded and the aptamer-functionalized fins were held in the top compartment [2]. If the fluid was changed to pH 3.2 buffer, the hydrogel contracted and the fins were pulled into the bottom compartment along with their load of thrombin [2]. Exposed to the low pH environment, the aptamers denatured and the thrombin was released [2]. After the thrombin was collected, pH 7.2 buffer could be pumped through the bottom compartment and the device would return to its resting state [2]. Not only was this able to specifically remove thrombin from a solution containing multiple proteins, the amount of thrombin removed could be increased by running the same solution through multiple cycles; from a solution containing 2 picomols of thrombin, over 95% was recovered after eight cycles [2].

## 6. pH-Responsive Nucleic Acid Motifs

**Figure 4:**
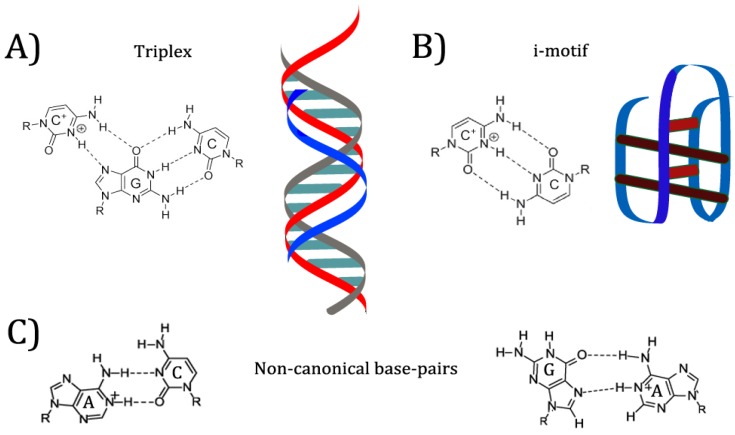
pH-responsive nucleic acid base-pair binding and motifs. (**A**) Illustration of C + GC triplex non-canonical base pair binding and the triplex binding motif. (**B**) Illustration of C + C non-canonical base pair binding and the i-motif, comprised of C + C bonds. (**C**) Left: Illustration of A + C non-canonical base pair binding. Right: Illustration of A + G non-canonical base pair binding.

In previous examples mentioned, the researchers took advantage of existing pH-responsive properties of the aptamers, and designed systems with those properties in mind. However, many of these systems required very acidic conditions to denature the aptamers and release the bound ligand. Research into DNA nanostructures has yielded many pH-responsive motifs that can be specifically designed to produce conformation switching at a desired pH value (see Figure 4) [8]. Nucleic acids can bind in many ways other than the canonical Watson-Crick orientation, including reverse Watson-Crick, Hoogsteen, and “wobble” base pairs [29]. Many of these interactions appear in common aptamer structures. For example, the G-quartet motif mentioned above is formed by Hoogsteen interactions between guanines from four separate strands, placing each guanine at right angles to the next on a linear plane [29]. When multiple G-quartets (often three) are stacked, the resulting helical structure is dubbed a G-quadruplex [30]. G-quadruplexes are commonly found in aptamers and other synthetic DNA nanostructures, as well as in mRNA and gene promoters [30]. Protonation of nucleobases can greatly increase the stability of some base pair mismatches [29]. Adenine and cytosine in particular are noted to become protonated at mildly acidic pH; depending on the structure of the folded nucleic acid, the pKa of protonation can be shifted up to neutrality, allowing mispairs to be stably incorporated into a structure [31]. Because protonation allows for different base pairings to become stable, changing the environmental pH can allow a DNA structure such as an aptamer to reversibly change conformation [29]. This conformation change has effects on properties such as target binding.

One commonly used pH-responsive DNA motif is the i-motif [32]. The i-motif consists of two intercalated duplexes, each held together by antiparallel C^+^C base pairing [32]. The pKa of isolated cytosine is 4.58 [33]. Under conditions of crowding or superhelicity, i-motifs can form at neutral pH; recent research has suggested that this structure regulates replication and transcription in vivo [34]. However, under most in vitro conditions, i-motifs are fully folded at pH 5–6 and unstable at neutral pH [33,34]. This pH dependence has made the i-motif popular for nanodevices designed to switch conformation under mildly acidic conditions [33]. In 2015, Bielecka and Juskowiak designed a fluorescent probe for pH monitoring that depended on i-motif formation, changing the protonation equilibrium of a fluorescent cytosine analogue and thereby quenching it under more acidic conditions [35]. I-motifs have also been incorporated into more complex nanostructures such as logic gates and motors [8]. Below, two aptamers are discussed that use the i-motif to incorporate pH-dependent binding.

The triplex structure is a second popular DNA motif that responds to pH [36]. Several combinations of bases are capable of forming a triplex, consisting of two strands in a Watson-Crick duplex and a third, additional strand bound to one of the other two through Hoogsteen or reverse Hoogsteen interactions [36]. The triplex motif has been used to drive structural change based on several triggers, one of the more common being strand displacement [37]. However, change in pH can also be used to stabilize or disrupt a triplex: in particular, the C^+^GC triplex motif is dependent on protonation of the cytosine on the third strand [36,37]. In 2014, Idili et al. were able to construct a DNA nanoswitch that “opened” or “closed” based on an intramolecular triplex structure [9]. By varying the proportion of C^+^GC triplets (pKa 4.5) and TAT triplets (pKa 10.2), they were also able to fine-tune the pH below which the switch was closed [9]. Two aptamers that use pH-controlled triplex formation to regulate target binding are discussed below.

Another pH-responsive motif is the A-motif, a parallel duplex consisting of A^+^A^+^ base pairs that forms at pH 3–4 [38]. It has been suggested that because the motif is stable at more acidic conditions than the i-motif or triplex, the A-motif may be useful for creating probes that are pH-sensitive below the 5.5–7.6 range most easily detected by i-motif probes [38].

In addition to the motifs discussed above, other non-canonical base pairs can be used to introduce pH-controlled aptamer binding. A^+^G, A^+^C, and C^+^C base pairs have all been noted to become much more stable when protonated [29]. In 2016, Fong et al. reported the successful in vitro selection of a pH-responsive nanoswitch using selection conditions comparable to SELEX, rather than deliberate alteration of a pre-existing structure [39]. The researchers found that the newly synthesized switch showed no sign of the previously reported pH-responsive i-motifs, poly-dA, or triplex structures [39]. Sequence analysis and mutation experiments suggested that the pH-responsive structure switching was controlled by C^+^A mispairs at key points [39].

## 7. Engineered pH-Responsive Aptamers

**Figure 5:**
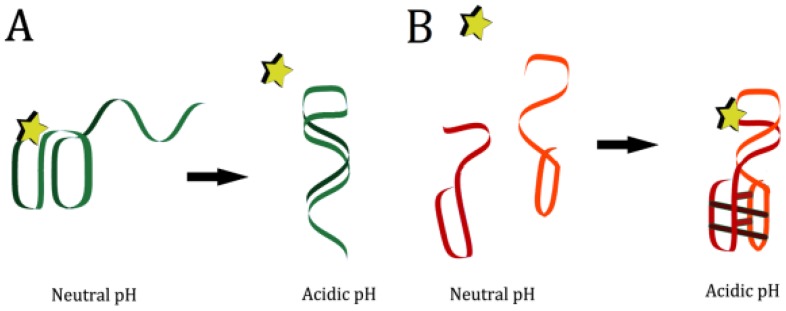
Methods of creating engineered pH-responsive aptamers. (**A**) An unstructured strand binds to the aptamer at a specific pH, disrupting the structure and preventing it from folding into target-binding conformation. (**B**) An aptamer in two halves is attached to two sides of a pH responsive motif. At high pH, the energetic cost of folding is too high for target to be bound. At low pH, when the motif forms, the aptamer halves are brought together, the energetic cost of aptamer folding is lowered, and target is bound.

There are several existing methods for engineering pH-responsive aptamer binding. The first strategy involves conformational change of the aptamer in response to pH: by incorporating pH-responsive motifs or non-canonical binding into the structure, the aptamer can be induced to fold into or out of binding conformation in response to specific pH levels [12,40]. A variation of this can be achieved using a pH-responsive strand that is partially complementary to the aptamer, controlling conformation through base pairing without altering the aptamer itself [37]. The second strategy requires a split aptamer, composed of two halves which associate to form the binding conformation [33]. Generally, the two strands are used 1:1 and in the high nanomolar to micromolar concentration range. Formation and denaturation of pH-responsive motifs can be used to control the proximity of attached aptamer halves, thereby controlling their association and target binding [3,33]. (See Figure 5).

In 2015, Porchetta et al. described a method to insert controllable pH-induced allostery into a wide range of DNA receptors [12]. As a demonstration, the researchers used a cocaine aptamer, previously observed to display little or no change in binding affinity over the pH 5.0 to pH 7.0 range [12]. To the 3′ end of the structure, they added a tail that, when protonated, was able to fold back on itself and form an intramolecular triplex structure with a portion of the aptamer [12]. This triplex was based on the CGC^+^ motif and showed a transition pKa (duplex to triplex) of 6.3 [12]. When the triplex formed, the aptamer was unable to fold completely or bind cocaine; the researchers observed that the affinity of the aptamer for cocaine could be “tuned” over two orders of magnitude when the pH was altered between pH 4.0 and 7.0 [12]. In a slightly basic environment (pH 8.0) where the triplex did not form, the observed affinity was only slightly lower than that of the unmodified aptamer, likely due to the portion of the tail that was able to form a non-pH-dependent duplex stem [12]. With fluorescent labeling, the researchers were able to observe “catch and release” of cocaine by the aptamer simply by changing the pH from 6.0 to 5.0 [12].

McConnell et al. investigated in 2016 a method of introducing pH dependence into an aptamer using the A^+^(anti)G(syn) motif, which would require less alteration of aptamers that already include a high proportion of G [40]. A thrombin aptamer was selected as a well-characterized example of the common G-quadruplex aptamer conformation [40]. Through rational design, a strand that would form terminal and central A^+^G mispairs was synthesized and tested; once it was confirmed that the strand would bind and disrupt quadruplex formation at pH 5.0, but not at pH 7.0, it was attached to the aptamer with a short linker [40]. The reversible change in conformation of the modified aptamer due to pH induced A^+^G binding was observed using fluorescent labeling [40]. When thrombin was added, “catch and release” by the aptamer due to G-quadruplex formation and disruption was observed as the pH of solution was cycled between 5 and 7 [40]. The unmodified aptamer showed no change in binding affinity between pH 5 and 7 [40]. The ability of the modified aptamer to bind and release thrombin decreased over multiple cycles; this was noted as likely due to the increasing ionic strength of the solution as the pH was switched [40].

In the same year, Del Grosso et al. designed a modular DNA nanoswitch able to respond to multiple inputs, including aptamer binding and pH [37]. One module used triplex binding, with a separate “activator” strand bound to a stem duplex; while the initial intent was for the activator to be added and removed via strand displacement, pH also functioned as a method to control binding [37]. When the triplex formed, it stabilized the structure of a split ATP-binding aptamer; when the triplex dissociated, the increased energetic cost meant that the two halves of the aptamer unfolded and the binding capacity was lost [37]. This allowed the nanoswitch to function as an AND gate, only able to bind ATP at low pH [37]. The system was also reversible, with a change in the order of the modules allowing the ATP aptamer to function as the switch to control triplex binding of the “activator” strand [37].

Also in 2016, Yan et al. used an i-motif to create a pH-responsive aptamer probe for detection of cancer cells in tissue samples [3]. Like the probe designed by Zhou et al. the following year, it was composed of a nucleolin-binding aptamer covalently attached to a fluorophore; unlike the previously described probe, the pH-responsive property was of the aptamer itself, rather than the fluorophore [3,25]. A cytosine-rich strand labeled with a quencher at the 5′ end was synthesized as a partial complement to a nucleolin-binding aptamer labeled with a fluorophore at the 3′ end [3]. At neutral pH, the two strands formed a duplex, preventing the aptamer from folding or binding and quenching the fluorescence from the probe [3]. At pH 6.0, however, the complement strand dissociated from the aptamer in favor of forming the i-motif structure [3]. This allowed the aptamer to fold into its target-binding conformation and the fluorescent signal to show [3]. When the probe was used to screen for tumour cells in sample tissues, researchers found that the slightly acidic environment of solid tumour cells was sufficient to dissociate the i-strand from the aptamer, allowing the fluorescent probe to bind to nucleolin expressed on the surface of cancer cells [3]. One downside of this system was that the probe was unstable at temperatures above 19 °C, and so could not be used in vivo without further alterations [3].

In 2017, Shi et al. created a nanodevice consisting of two halves each of an aptamer and an i-motif joined to opposite ends of a three-way-junction and a four-way-junction branched nanostructure [33]. One half of the aptamer was functionalized with a fluorophore while the other half was attached to a quencher, allowing the folding and binding to be detected [33]. It was observed that at pH 8, there was no fluorescence quenching, indicating that the aptamer was unable to fold or bind while the two halves were bound to separate branched structures [33]. At pH 5, the two halves of the i-motif were able to associate, bringing together the two branched structures and resulting in a small amount of quenching as the aptamer halves were brought into closer proximity; upon addition of ATP, a sharp decrease in fluorescence was observed, as the aptamer associated and bound ATP, bringing the fluorophore and quencher together [33]. Returning the pH to 8.0 caused the aptamer to dissociate and release the ATP [33].

## 8. Conclusions

While aptamers have been effectively incorporated into pH-activatable systems, and tools exist for manipulating the binding capabilities of aptamers, the applications of engineered pH-activatable aptamers are still largely unexplored. As an expansion of the drug-delivery systems described above, aptamers that activate at weakly acidic pH could act as an extra layer of control for the drug delivery system, ensuring that the nanoparticle is only taken up by cells in the acidic tumour microenvironment and preventing off-target toxicity. Another avenue worthy of exploration could involve controlling the pH at which an aptamer binds or releases its cargo to improve systems such as the graphene oxide and hydrogel capture systems described above, or in affinity purifications of proteins that would denature at extreme pH. AND switches responding to both pH and ligand presence have further applications in nanotechnology, acting as logic gates for fine control of nanomachines, and should be expanded to new targets that could be relevant “fuels” for nanomachines. It is important to note that the majority of examples described herein explore activation at low pH as an inherent consequence of the pKas of the DNA bases. New applications examining base-induced switching could open up interesting possibilities. There is great untapped potential within the field of pH-activatable aptamer systems; better understanding of the mechanisms behind pH-responsive binding and effective methods of introducing it could facilitate the rational design of these systems for numerous applications.
